# Effect of milk fat globule membrane supplementation on motor unit adaptation following resistance training in older adults

**DOI:** 10.14814/phy2.14491

**Published:** 2020-06-29

**Authors:** Kohei Watanabe, Aleš Holobar, Aya Tomita, Yukiko Mita

**Affiliations:** ^1^ Laboratory of Neuromuscular Biomechanics School of International Liberal Studies Chukyo University Nagoya Japan; ^2^ Faculty of Electrical Engineering and Computer Science University of Maribor Maribor Slovenia; ^3^ Department of Human Nutrition School of Life Studies Sugiyama Jogakuen University Nagoya Japan

**Keywords:** aging, motor unit identification, multichannel surface electromyography, nutritional supplementation

## Abstract

This study aimed to investigate the effect of milk fat globule membrane (MFGM) supplementation on motor unit adaptation following resistance training in older adults. Twenty‐five older males and females took MFGM (*n* = 12) or a placebo (PLA; *n* = 12) while performing 8 weeks of isometric knee extension training. During the training, the motor unit firing pattern during submaximal contractions, muscle thickness, and maximal muscle strength of knee extensor muscles were measured every 2 weeks. None of the measurements showed significant differences in muscle thickness or maximal muscle strength (MVC) between the two groups (*p* > .05). Significant decreases in motor unit firing rate following the intervention were observed in PLA, that is, 14.1 ± 2.7 pps at 0 weeks to 13.0 ± 2.4 pps at 4 weeks (*p* = .003), but not in MFGM (14.4 ± 2.5 pps to 13.8 ± 1.9 pps). Motor unit firing rates in MFGM were significantly higher than those in PLA at 2, 4, 6, and 8 weeks of the intervention, that is, 15.1 ± 2.3 pps in MFGM and 14.5 ± 3.3 pps in PLA at 70% of MVC for motor units recruited at 40% of MVC at 6 weeks (*p* = .034). Significant differences in firing rates among motor units with different recruitment thresholds were newly observed following the resistance training intervention in MFGM, indicating that motor unit firing pattern is changed in this group. These results suggest that motor unit adaptation following resistance training is modulated by MFGM supplementation in older adults.

## Graphical abstract

The positive effect of milk fat globule membrane (MFGM) supplementation with exercise on age‐related dysfunctions in neuromuscular system had been reported in animal and human studies. This study found that MFGM supplementation with exercise can modify adaptations in motor unit firing pattern in older adults. Whereas the same exercise regimen was applied, different neural adaptation may be able to induce by ingestion of MFGM in older adults.

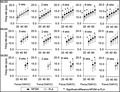

## 1 INTRODUCTION

Benefits of resistance training to prevent age‐related muscle strength loss have been widely recognized (Fragala et al., 2019). Also, it has been suggested that some nutritional supplementations can enhance or modify the adaptations following resistance training (Dickinson, Volpi, & Rasmussen, 2013). For example, recent studies showed the enhancement of increases in muscle mass and strength following resistance training with protein supplementation in older adults (Cermak, Res, de Groot, Saris, & van Loon, 2012; Liao et al., 2017). Protein supplementation may contribute to the improvement of muscular adaptations such as muscle hypertrophy via muscle protein synthesis. On the other hand, it has been reported that neural adaptations markedly contribute to improvements in muscle strength induced by resistance training when compared with muscular adaptations in older adults (Hakkinen et al., 1998; Kamen & Knight, 2004; Moritani & deVries, 1980). For example, we had already reported that significant correlation between maximal muscle strength and motor unit firing rates in knee extensor muscles is observed in older adults, but not in young adults (Watanabe et al., 2016). Kamen and Knight (2004) demonstrated that maximal motor unit firing rate increased 15% in young adults and 49% in older adults following 6 weeks resistance exercise training program (Kamen & Knight, 2004). These results mean that contribution and trainability of neural factors to muscle strength may be greater in older adults. However, targets for preventing age‐related muscle strength loss using nutritional supplementations combined with resistance training are primarily muscular and not neural adaptations, which would be more trainable in older adults.

Recently, some studies showed the possibility of regulating neural adaptations by nutritional supplementation in conjunction with exercise. Milk fat globule membrane (MFGM) is a membrane that surrounds the triglyceride core of each fat globule and contains sphingomyelin, which is a type of sphingolipid found in animal cell membranes, especially in the membranous myelin sheath that surrounds some nerve cell axons. Dietary ingestions of MFGM and sphingomyelin induce an increase in gene expression promoting neuromuscular junction formation in aging mice following voluntary running exercise and myelination in the central nervous system of developing rats with experimental inhibition of activity (Haramizu et al., 2014; Oshida, Shimizu, Takase, Tamura, & Yamashiro, 2003; Yano, Haramizu, Ota, Minegishi, & Shimotoyodome, 2019; Yano, Minegishi, Sugita, & Ota, 2017). These results suggest that dietary MFGM or sphingomyelin supplementation can counteract the aging‐related dysfunctions in neural systems associated with denervation or demyelination at neuromuscular junctions. Moreover, other research showed that MFGM supplementation elicited improvements in motor functions associated with muscle strength and agility following exercise interventions in older adults (Minegishi, Ota, Soga, & Shimotoyodome, 2016; Ota, Soga, Hase, & Shimotoyodome, 2015; Soga, Ota, & Shimotoyodome, 2015). They also showed that MFGM ingestion increases the surface electromyography (EMG) amplitude and conduction velocity following improvement of motor functions (Minegishi et al., 2016; Soga et al., 2015), which may reflect an increase in motor unit firing or a change in recruitment patterns due to the ingestion of MFGM. However, the detailed effects of MFGM or sphingomyelin ingestions on neural activation, such as the motor unit firing pattern, have not been directly investigated in humans.

The motor unit decomposition technique with high‐density surface electromyography was developed to assess neural activation in humans (Farina, Merletti, & Enoka, 2014; Holobar, Farina, Gazzoni, Merletti, & Zazula, 2009; Merletti, Holobar, & Farina, 2008). Since this technique is noninvasive and detects a relatively larger number of motor units compared with intramuscular electromyography, it may be useful for repetitive measurements such as in intervention studies. In fact, recent studies using this novel technique have provided important new knowledge of neural adaptations following resistance training (Del Vecchio et al., 2019; Martinez‐Valdes, Farina, Negro, Del Vecchio, & Falla, 2018; Vila‐Cha, Falla, Correia, & Farina, 2012; Vila‐Cha, Falla, & Farina, 2010). Its application may be more useful for studies in older adults, since it is well‐known that neural adaptations mainly contribute to improvement of muscle strength following resistance training in older adults (Hakkinen et al., 1998; Kamen & Knight, 2004; Moritani & deVries, 1980).

The purpose of this study was to investigate the effect of MFGM supplementation on the motor unit firing pattern following resistance training in older adults. Based on previous studies reporting that exercise and MFGM ingestion induce changes in neuromuscular junctions in mice (Haramizu et al., 2014; Oshida et al., 2003; Yano et al., 2017, 2019) and improvements in the motor function with an increase in surface EMG amplitude and conduction velocity in humans (Minegishi et al., 2016; Soga et al., 2015), neuromuscular activation should be improved following exercise with MFGM ingestion. We thus hypothesized that MFGM ingestion induces an increase in motor unit firing rates at the same absolute force and then changes adaptations in the motor unit firing pattern during resistance training.

## 2 MATERIALS AND METHODS

### 2.1 Participants

Twenty‐five healthy older males and females (mean age ± *SD*: 73.1 ± 5.9 years, range: 63–84 years) participated in this study. The participants who were restricted any exercises by medical doctors were excluded when the participants were recruited. The subjects gave written informed consent for the study after receiving a detailed explanation of the purposes, potential benefits, and risks associated with participation. All procedures used in this study were approved by the Research Ethics Committee of Chukyo University (2018‐008) and conducted in accordance with the Declaration of Helsinki, and the study design was submitted to the University Hospital Medical Information Network in Japan (UMIN000033932).

### 2.2 Experimental design

Participants were randomly divided into two groups: MFGM (*n* = 12) and placebo (PLA; *n* = 12), based on the age, sex, and muscle strength at the baseline. Detailed characteristics of participants in MFGM and PLA groups are presented in Table 1.

**TABLE 1:** Characteristics of participants

Group	MFGM	PLA	MFGM versus PLA
*n*	12	12	
*n* of males	7	7	
Age (years)	72.3 ± 5.2	73.9 ± 6.6	*p* = .320
Height (cm)	159.1 ± 7.1	160.5 ± 10.3	*p* = .689
Body mass (kg)	56.0 ± 8.1	58.9 ± 11.4	*p* = .728

Note

No significant differences were detected between MFGM and PLA groups.

All participants trained their knee extensor muscles by isometric knee extension resistance training for 8 weeks. During the intervention, the participants performed the resistance training twice a week. A training day consisted of three sets of five isometric contractions during unilateral knee extension for both left and right legs with a rest interval of 60–120 s. The contraction intensity was set at greater than 80% of their maximal voluntary contraction (MVC) torque, and the performed and target torques were shown on the monitor of a personal computer as visual feedback. Each contraction consisted of a 10‐s resting phase, 1‐s increasing phase to the target torque, and a 4‐s sustained phase at or exceeding the target torque. During contractions, the experimenter requested that the subjects count to 5 s aloud in order to prevent breath‐hold and provided verbal encouragements. The target torque was updated when MVC increased after measurements conducted every 2 weeks. The dynamometer system and posture of the subjects for training were the same as for MVC measurements (see below).

Participants took tablet‐type foods including MFGM or PLA in addition to daily meals every day during the intervention. MFGM was produced from raw milk by centrifugation and ultrafiltration. MFGM contained abundant sphingomyelin. The tablets were produced by direct compression of the mixture. Detailed process of centrifugation steps was described in Ota et al. (2015). Participants took MFGM (1 g/day) or PLA (1 g/day) tablets in addition to daily meals every day during the intervention. The composition of MFGM tablets containing sphingomyelin at 43.6 mg/g was: 22.5% protein, 13.2% fat, 59.5% carbohydrate, 3.0% ash, and 1.8% moisture. The PLA tablet was prepared using milk powder instead of MFGM. The composition of PLA tablets was: 11.6% protein, 12.7% fat, 71.0% carbohydrate, 3.3% ash, and 1.4% moisture. Sphingomyelin was not detected in PLA tablets. These supplementations were the same as used in previous studies showing the enhancement of motor performance with exercise intervention in older adults (Minegishi et al., 2016; Ota et al., 2015; Soga et al., 2015). The tablets for MFGM and PLA had a similar shape, flavor, and weight. A randomized, double‐blind, placebo‐controlled treatment was conducted for supplementation.

A dietary survey was performed at 1 and 8 weeks by a nutritionist with a license from the Ministry of Health, Labour and Welfare in Japan and they used a brief‐type self‐administered diet history questionnaire (BDHQ; Kobayashi et al., 2012). Since this questionnaire asks about the history of dietary habits for a month, the results of BDHQ in the first and eighth weeks reflected daily diets before and during the intervention, respectively. We quantified total energy, protein, fat, and carbohydrate in daily diets before and during the intervention. Intakes of low‐fat milk and milk were also calculated and assessed based on this questionnaire.

The participants came to our laboratory at least 1 week prior to the first day of intervention to familiarize themselves with the instrumentation and motor tasks. Muscle strength, motor unit firing pattern, and anthropometric tests were performed every 2 weeks during the intervention: on the first day before the intervention commenced (0 weeks), and at 2, 4, 6, and 8 weeks after beginning the intervention.

### 2.3 Muscle strength and anthropometric tests

The MVC and rate of force development (RFD) during isometric knee extension of the right leg were measured at 0, 2, 4, 6, and 8 weeks using a custom‐made dynamometer (Takei Scientific Instruments Co., Ltd.) with force transducer (LU‐100KSE; Kyowa Electronic Instruments). During the measurement, both hip and knee joint angles were set at 90° (180° corresponds to full extension) and the distal part of the shank of the right leg was fixed to the force transducer in the dynamometer.

The participants performed at least two MVC trials with a ≥2‐min rest interval between them. An MVC trial included a gradual increase in the knee extension force to maximum effort in 2–3 s, and the plateau phase at maximum effort was maintained for 2–3 s with a verbal count given at 1‐s intervals. The highest MVC force was chosen from MVC trials and used to calculate MVC torque as a product of the knee extension force and distance between the estimated rotation axes for the knee joint in the sagittal plane. For RFD, the participants performed two sets of five trials. Rest intervals between trials and sets were 10 s and 2 min, respectively. The participants were instructed to perform the greater force as quickly as possible following a verbal count from the investigator during RFD trials.

As anthropometric data, the body mass, whole body muscle mass, and lower limb muscle mass were obtained from body impedance measurements (InBody270, InBodyJapan Inc.) at 0, 4, and 8 weeks. We also measured the thickness of knee extensor muscles of the right leg by ultrasonographic imaging (LOGIQ *e* Premium, GE Healthcare). Transversal B‐mode images were taken at 50% of the distance between the head of the greater trochanter and inferior lateral edge of the patella, at the location of the center of the electrode grid for surface EMG recording. The vertical distance between the superficial edge of the vastus lateralis (VL) muscle and superficial edge of the femur was measured as the muscle thickness with image analysis software (Image J, National Institutes of Health). This muscle thickness includes the VL and vastus intermedius muscles. A single operator blinded to information on subject groups and test periods carried out this analysis. These measurements were also used in our previous studies (Watanabe et al., 2016, 2019).

We also estimated the level of daily physical activity by International Physical Activity Questionnaire (IPAQ short, Japanese version) during the intervention. Questions on the duration (minutes) and frequency (days/week) of walking, moderate‐intensity activities, and vigorous‐intensity activities were asked in the questionnaire. Walking in this questionnaire includes a walk during work and daily living and walking as a hobby and/or exercise. As examples of activities at each intensity, carrying light cargo, playing with children, swimming slowly, playing tennis in doubles games, and playing golf without electrical carts were categorized as examples of moderate‐intensity, and carrying heavy cargo, climbing a hill by bicycle, jogging, and playing tennis in singles games were categorized as examples of vigorous‐intensity. Based on the answers regarding the duration and frequency of each physical activity, energy expenditures were calculated for walking, moderate‐intensity activities, and vigorous‐intensity activities and total activities in a week during the intervention. Metabolic equivalent (METs) were applied for this calculation and 3.3, 4.0, and 8.0 METs were used for walking, moderate‐intensity activities, and vigorous‐intensity activities, respectively (Craig et al., 2003).

### 2.4 Multi‐channel surface EMG recording and motor unit decomposition

For assessment of motor unit firing patterns, multi‐channel surface EMG signals were recorded from the VL muscle of the right leg during submaximal ramp contractions at 0, 2, 4, 6, and 8 weeks. Participants performed isometric knee extension ramp contractions from 0% to 30% of MVC (Ramp30) and 0% to 70% of MVC (Ramp70). The MVC torque used for calculation of the target torque in all periods was the same as for MVC measured at 0 weeks, which means that the participants exerted the same absolute value of torque at 0, 2, 4, 6, and 8 weeks. The torque measurement system and posture during these submaximal contractions were the same as those during MVC. Two trials were performed for each task with a >2‐min rest interval. Ramp30 consisted of a 15‐s increasing phase from the baseline to 30% of the MVC force level with a 2% MVC/s rate of force increase and 15‐s sustained phase at 30% of the MVC force level. Ramp70 consisted of a 14‐s increasing phase from the baseline to 70% of the MVC force level with a 5% MVC/s rate of force increase and 5‐s sustained phase at 70% of the MVC force level. Durations of ramp‐up and sustained phases in these motor tasks were determined from the results of preliminary experiments in order to maximize the numbers of detected motor units. Out of two trials, the trial with the smaller error between the target and actual forces was selected for analysis of Ramp30 and Ramp70, respectively.

We used a semi‐disposable adhesive grid of 64 electrodes with a 1‐mm diameter and 8‐mm interelectrode distance (ELSCH064NM2, OT Bioelectronica) for recording the multi‐channel surface EMG signal. The electrodes were organized in 13 rows and 5 columns with one missing electrode in the upper left corner. The method used for determining the electrode location was as previously described (Watanabe et al., 2016, 2019). To set the electrode grid at the same position in the different periods during intervention, the electrode locations were determined using bone markers. The mid‐point of the line between the head of the greater trochanter and inferior lateral edge of the patella was used as the center of the electrode grid, and the line was also used to determine the direction of electrode grids, whereby columns of electrodes were aligned along it. A reference electrode (WS1, OT Bioelectronica) was placed at the right knee. Monopolar surface EMG signals were recorded with a band‐pass filter (10–450 Hz), and amplified by a factor of 500, sampled at 2,048 Hz, and converted to a digital form by a 16‐bit analog‐to‐digital converter (Quattrocento, OT Bioelectronica). The signal from the force transducer was synchronized with this analog‐to‐digital converter.

Recorded monopolar surface EMG signals were transferred to analysis software (MATLAB R2018a, MathWorks GK), and individual motor units were identified by the Convolution Kernel Compensation (CKC) technique using DEMUSE software (Holobar et al., 2009; Holobar & Zazula, 2004, 2008; Merletti et al., 2008). We followed the decomposition procedure previously and extensively validated based on signals from various skeletal muscles (Farina, Holobar, Merletti, & Enoka, 2010; Gallego, Dideriksen, Holobar, Ibanez, Glaser, et al., 2015; Gallego, Dideriksen, Holobar, Ibanez, Pons, et al., 2015; Holobar et al., 2009; Watanabe et al., 2016, 2018; Yavuz et al., 2015). The pulse‐to‐noise ratio (PNR), introduced by Holobar, Minetto, and Farina (2014), was used as an indicator of the motor unit identification accuracy (Holobar et al., 2014), and only motor units with PNR > 30 dB (corresponding to an accuracy of motor unit firing identification >90%) were used for further analysis; all other motor units were discarded (Holobar et al., 2014). After decomposition, discharge patterns of individual motor units were independently examined by two experienced investigators. Discharge times for individual motor units were used to calculate instantaneous motor unit firing rates. We excluded discharges with inter‐discharge intervals <33.3 or >250 ms, since firing rates calculated from this range of inter‐discharge intervals are unusually high (>30 Hz) or low (<4 Hz) for the VL muscle (Adam & De Luca, 2005; Holobar et al., 2009; Watanabe et al., 2013, 2016; Welsh, Dinenno, & Tracy, 2007). These procedures were the same as those used in our previous studies (Watanabe et al., 2016, 2018, 2019).

Median values of firing rates of individual motor units were calculated from instantaneous firing rates every 5% of MVC for Ramp30 and 10% of MVC for Ramp70, and those with >30% coefficient of variation were excluded from further analysis (Fuglevand, Winter, & Patla, 1993). Detected motor units were divided into three groups by the recruitment force: motor units recruited at <10% (MU10), 10%–20% (MU20), and 20%–30% (MU30) of MVC for Ramp30 and at <20% (MU20), 20%–40% (MU40), and 40%–60% (MU60) of MVC for Ramp70. Mean motor unit firing rates were calculated from all motor units from subjects of the same group for each motor unit group with different recruitment thresholds every 5% and 10% of MVC for Ramp30 and Ramp70 in different periods. This procedure to calculate the motor unit firing rate was the same as in our previous studies (Watanabe et al., 2016, 2018, 2019).

### 2.5 Statistics

The results are reported as the mean ± *SD*. Since our results included non‐normal distributed data and were measured based on small samples, the present study used nonparametric statistical tests. At 0 weeks, the age, height, body mass, whole body muscle mass, and lower limb muscle mass measured by the body impedance method, muscle tissue thicknesses measured by ultrasonography, MVC, and RFD were compared between the groups by the Mann–Whitney test. The body mass, whole body muscle mass, lower limb muscle mass, muscle tissue thicknesses, MVC, and RFD in all measured periods were analyzed by the Friedmann test to examine the effect of intervention in each group. When significant effects of intervention were observed, the Bonferroni–Dunn test was used to compare the values at 2, 4, 6, and 8 weeks with the value at 0 weeks (Pereira, Afonso, & Medeiros, 2015). Also, these values in each period, nutritional parameters before and during the intervention, and levels of physical activity during the intervention were compared between the groups by the Mann–Whitney test.

For motor unit firing rates of each motor unit group, the effects of intervention and nutritional supplementation were assessed by the Kruskal–Wallis test with Dunn's test for each participants’ group and the Mann–Whitney test between MFGM and PLA groups. To assess the relationship of the firing rate among the different motor unit groups for quantification of the motor unit firing pattern, the motor unit firing rate at each force level was also compared among the motor unit groups with different recruitment forces by the Kruskal–Wallis test with Dunn's test. Since this study used multiple comparisons, the level of significance was modified by Bonferroni correction (the modified level of significance was 0.05/ the numbers of comparisons for each analysis) to minimize the family wise error rate and to keep statistical power (Vincent, 2005). Therefore, for example, the level of significance in the post hoc test to compare the variables among the periods was set at 0.0125 (0.05/4) when the variables at 2, 4, 6, and 8 weeks are compared with 0 weeks (4 pairs). The level of significance in comparisons between two groups was set at 0.05. Statistical analysis was performed using SPSS (version 21.0, SPSS) and MATLAB (R2018a, MathWorks GK).

Statistical power was calculated for comparisons between the groups for each period and among the periods for each group in the variables used in this study (Vincent, 2005).

## 3 RESULTS

### 3.1 Anthropometric and muscle strength tests and others

At 0 weeks, there were no significant differences in age (*p* = .320), height (*p* = .689), body mass (*p* = .728), whole body muscle mass (*p* = .852), lower limb muscle mass (*p* = .769), muscle tissue thicknesses (*p* = .320), MVC (*p* = .713), or RFD (*p* = .256) between MFGM and PLA groups (Tables 1 and 2). Significant increases in MVC at 4 (*p* = .001), 6 (*p* = .001), and 8 weeks (*p* = .003) and in RFD at 8 weeks (*p* = .003) compared with 0 weeks were observed in the PLA group (Table 2).

**TABLE 2:** Results of anthropometric parameters and motor functions

Group	MFGM	Post hoc versus 0 weeks	PLA	Post‐hoc versus 0 weeks	MFGM versus PLA
Body mass (kg)					
0 weeks	56.0 ± 8.1		58.9 ± 11.4		*p* = .728
4 weeks	56.5 ± 8.4		59.3 ± 11.4		*p* = .769
8 weeks	56.5 ± 8.6		59.8 ± 11.8		*p* = .650
Whole body muscle mass (kg)					
0 weeks	22.7 ± 5.0		23.4 ± 5.5		*p* = .852
4 weeks	22.9 ± 5.2		23.6 ± 5.6		*p* = .728
8 weeks	22.8 ± 5.1		23.6 ± 5.6		*p* = .728
Lower limb muscle mass (kg)					
0 weeks	12.7 ± 2.7		13.3 ± 3.6		*p* = .769
4 weeks	12.8 ± 2.8		13.3 ± 3.7		*p* = .728
8 weeks	12.7 ± 2.7		13.3 ± 3.6		*p* = .728
Muscle thickness (cm)					
0 weeks	3.53 ± 0.56		3.22 ± 0.0		*p* = .320
2 weeks	3.53 ± 0.71		3.34 ± 0.73		*p* = .538
4 weeks	3.52 ± 0.76		3.28 ± 0.72		*p* = .376
6 weeks	3.56 ± 0.88		3.37 ± 0.76		*p* = .894
8 weeks	3.51 ± 0.63		3.39 ± 0.75		*p* = .611
MVC (Nm)					
0 weeks	118.0 ± 42.6		109.8 ± 38.7	—	*p* = .713
2 weeks	124.4 ± 43.6		123.7 ± 45.5	*p* = .014	*p* = .977
4 weeks	123.4 ± 43.9		126.9 ± 44.8	* *p* = .001	*p* = .932
6 weeks	122.1 ± 42.8		125.3 ± 40.3	* *p* = .001	*p* = .932
8 weeks	123.3 ± 40.2		126.3 ± 43.2	* *p* = .003	*p* = .932
RFD (Nm/s)					
0 weeks	807.5 ± 472.2		586.1 ± 260.4	—	*p* = .256
2 weeks	815.1 ± 329.8		650.1 ± 284.6	*p* = .233	*p* = .319
4 weeks	761.8 ± 292.0		673.1 ± 367.9	*p* = .020	*p* = .410
6 weeks	768.1 ± 286.8		640.7 ± 296.7	*p* = .081	*p* = .198
8 weeks	802.9 ± 377.6		717.7 ± 327.9	* *p* = .003	*p* = .713

Note

No significant differences were detected between MFGM and PLA groups.

* *p* < .0125 versus 0 weeks.

There were no significant differences in nutritional parameters, that is, total energy (*p* = .186 and .295), protein (*p* = .205 and .247), fat (*p* = .320 and .689), carbohydrate (*p* = .137 and .574), milk (*p* = .936 and .769), or low‐fat milk (*p* = .611 and .295), between the MFGM and PLA groups before or during the intervention (Table 3). Total energy (*p* = .012) and carbohydrate (*p* = .006) levels in the MFGM group and milk consumption (*p* = .017) in PLA were significantly increased and decreased from before to during the intervention (Table 3).

**TABLE 3:** Results of diet survey using a brief‐type self‐administered diet history questionnaire (BDHQ)

Group	MFGM	versus Before	PLA	versus Before	MFGM versus PLA
Total energy (kcal)					
Before	1,789 ± 654	—	2,099 ± 568	—	*p* = .186
During	2,145 ± 658	* *p* = .012	2,186 ± 483	*p* = .695	*p* = .295
Protein (g)					
Pre	77 ± 35	—	85 ± 21	—	*p* = .205
During	87 ± 46	*p* = .500	83 ± 17	*p* = 1.000	*p* = .247
Fat (g)					
Pre	57 ± 19	—	64 ± 18	—	*p* = .320
During	59 ± 23	*p* = .695	60 ± 17	*p* = .695	*p* = .689
Carbohydrate (g)					
Pre	222 ± 88	—	291 ± 101	—	*p* = .137
During	291 ± 101	* *p* = .006	301 ± 88	*p* = .308	*p* = .574
Milk (g)					
Pre	114 ± 80	—	127 ± 83	—	*p* = .936
During	97 ± 75	*p* = .237	94 ± 94	* *p* = .017	*p* = .769
Low‐fat milk (g)					
Pre	55 ± 128	—	46 ± 66	—	*p* = .611
During	57 ± 126	*p* = 1.000	70 ± 73	*p* = .138	*p* = .295

Note

No significant differences were detected between MFGM and PLA groups.

* *p* < .05 versus before.

Regarding levels of physical activity, while no significant differences were observed between the MFGM and PLA groups (*p* > .05), walking (*p* = .114), moderate‐intensity activities (*p* = .089) and total activities (*p* = .101) tended to be greater in the PLA than MFGM group (Table 4).

**TABLE 4:** Levels of daily physical activity calculated from International Physical Activity Questionnaire

Group	MFGM	PLA	MFGM versus PLA
Walking (kcal)	1,094 ± 1,072	2,163 ± 2,271	*p* = .114
Moderate‐intensity activities (kcal)	421 ± 493	2,178 ± 3,139	*p* = .089
Vigorous‐intensity activities (kcal)	353 ± 736	354 ± 749	*p* = .843
Total activities (kcal)	1,868 ± 1,367	4,595 ± 4,959	*p* = .101

Note

No significant differences were detected between MFGM and PLA groups.

### 3.2 Motor unit firing patterns

In this study, 679 and 555 motor units in the MFGM group and 831 and 501 motor units in the PLA group were detected and used for analysis during Ramp30 and Ramp70, respectively.

**FIGURE 1:**
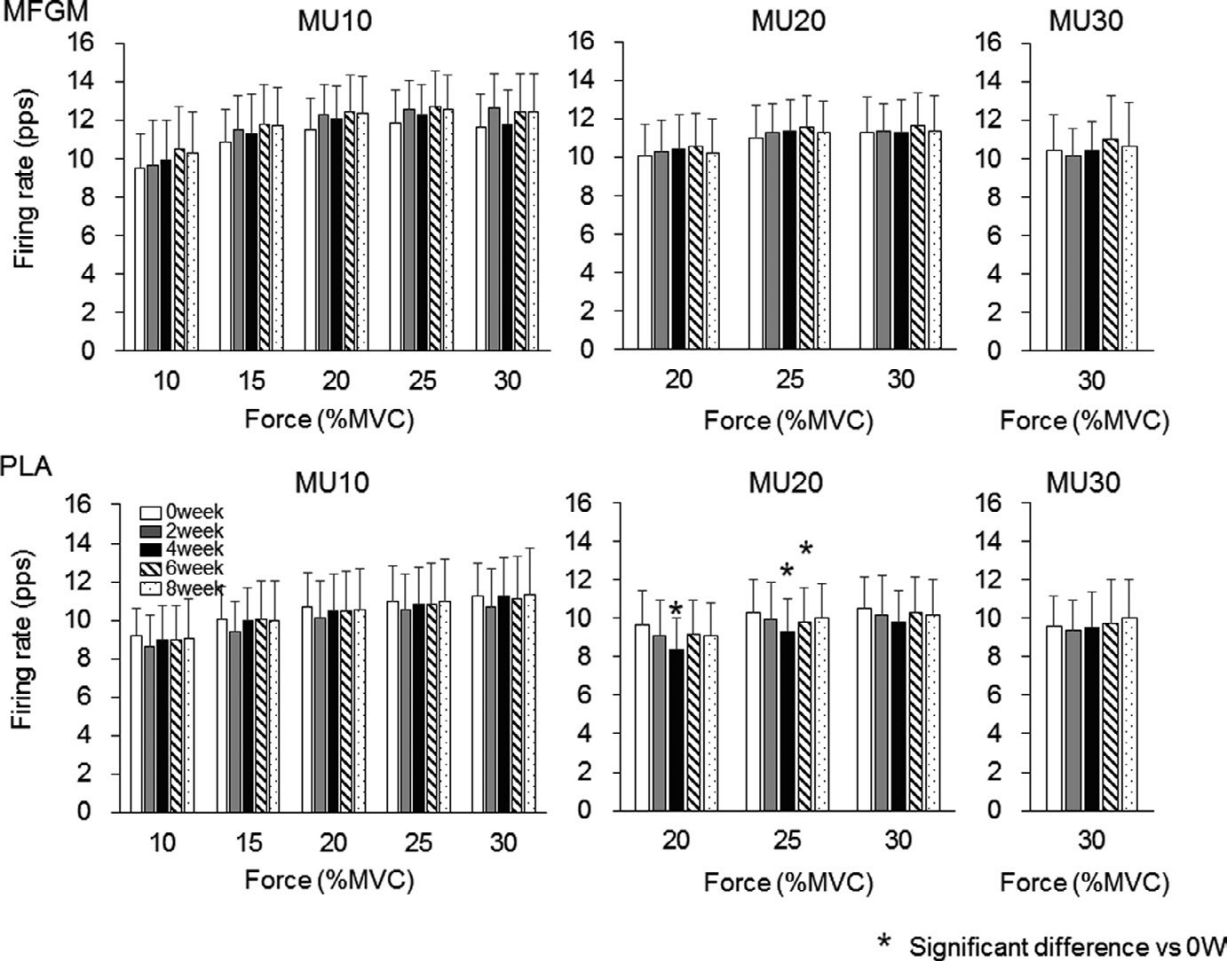
Motor unit firing rates during Ramp30 for the groups with ingestions of milk fat globule membrane (MFGM; upper panels) and placebo (PLA; lower panels). MU10: motor units recruited at <10% of maximal voluntary contraction (MVC), MU20: motor units recruited at 10%–20% of MVC, MU30: motor units recruited at 20%–30% of MVC. The symbol * indicates significant differences (*p* < .0125) when compared with 0 weeks

**FIGURE 2:**
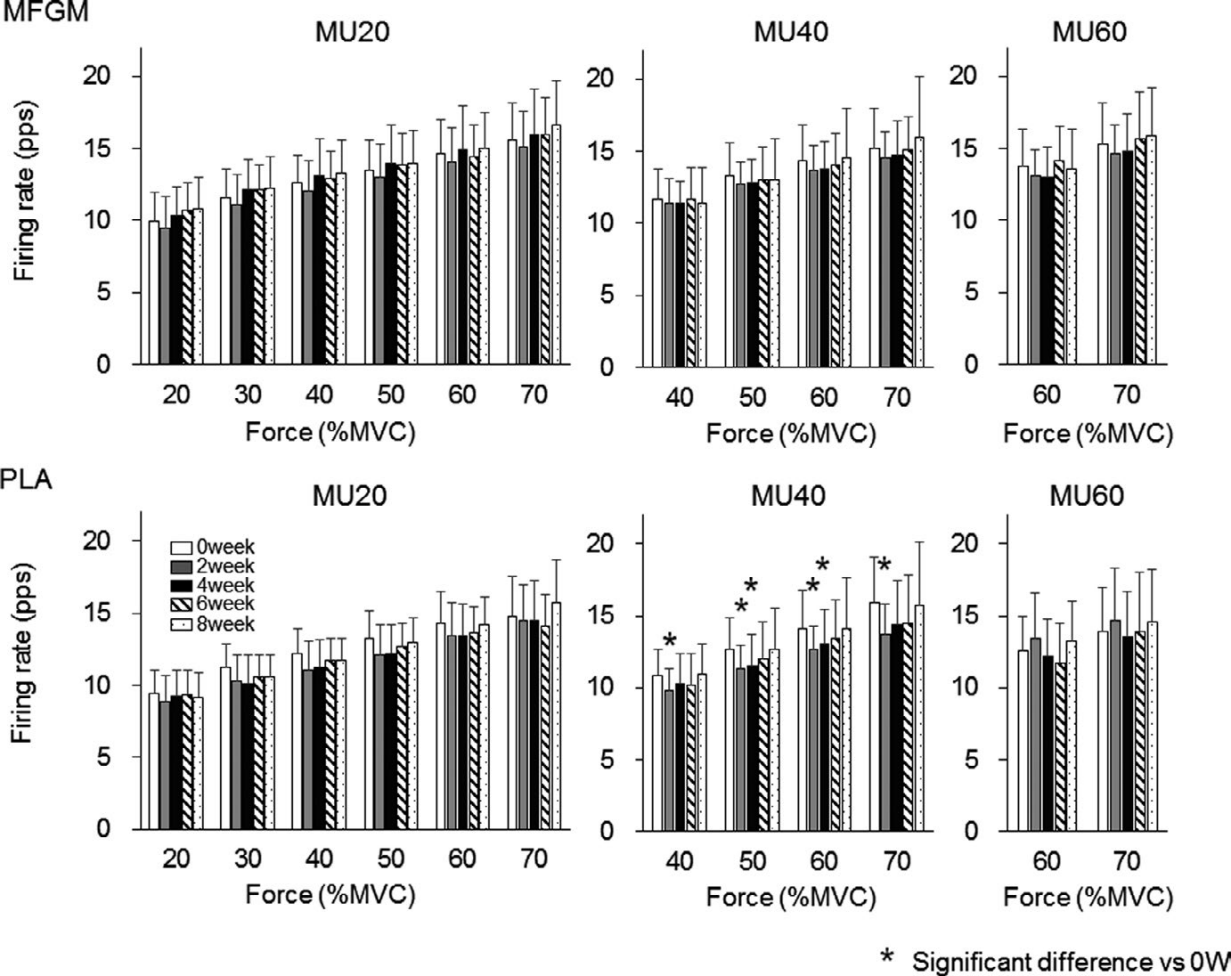
Motor unit firing rates during Ramp70 for the groups with ingestions of milk fat globule membrane (MFGM; upper panels) and placebo (PLA; lower panels). MU20: motor units recruited at <20% of maximal voluntary contraction (MVC), MU40: motor units recruited at 20%–40% of MVC, MU60: motor units recruited at 40%–60% of MVC. The symbol * indicates significant differences (*p* < .0125) when compared with 0 weeks

Motor unit firing rates in the MFGM group were not significantly changed during the intervention in MU10 (*p* = .477, .766, .791, .584, and .289 at 10%, 15%, 20%, 25%, and 30% of MVC), MU20 (*p* = .851, .771, and .636 at 20%, 25%, and 30% of MVC), and MU30 (*p* = .446 at 30% of MVC) for Ramp30 (Figure 1) and in MU20 (*p* = .481, .918, .654, .559, .534, and .324 at 20%, 30%, 40%, 50%, 60%, and 70% of MVC), MU40 (*p* = .956, .805, .599, and .412 at 40%, 50%, 60%, and 70% of MVC), and MU60 (*p* = .483 and .099 at 60% and 70% of MVC) for Ramp70 (Figure 2). For Ramp30, firing rates of MU20 were significantly decreased at 20% of MVC in 4 weeks (8.4 ± 1.6 pps, *p* = .001) and at 25% of MVC in 4 (9.3 ± 1.7 pps, *p* = .001) and 6 weeks (9.8 ± 1.8 pps, *p* = .004) when compared with those at 0 weeks (9.7 ± 1.8 and 10.3 ± 1.7 pps at 20% and 25% of MVC) in the PLA group (Figure 1). For Ramp70, firing rates of MU40 were significantly decreased at 40% of MVC in 2 weeks (9.8 ± 1.5 pps, *p* = .004), at 50% and 60% of MVC in 2 (11.3 ± 1.7 and 12.6 ± 1.7 pps, *p* = .001 and .004) and 4 weeks (11.5 ± 2.2 and 13.0 ± 2.4 pps, *p* = .004 and .003), respectively, and at 70% of MVC in 2 weeks (13.7 ± 2.2 pps, *p* = .005) when compared with those at 0 weeks (11.7 ± 2.1, 13.3 ± 2.3, 14.1 ± 2.7, and 15.2 ± 2.8 pps at 40%, 50%, 60%, and 70% of MVC) in the PLA group (Figure 2).

**FIGURE 3:**
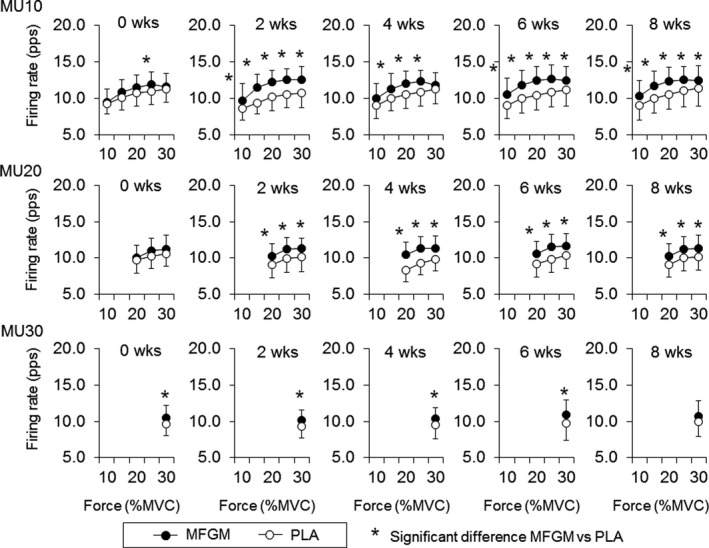
Comparisons of motor unit firing rates during Ramp30 between the groups with ingestions of milk fat globule membrane (MFGM; filled circles) and placebo (PLA; open circles). MU10: motor units recruited at <10% of maximal voluntary contraction (MVC), MU20: motor units recruited at 10%–20% of MVC, MU30: motor units recruited at 20%–30% of MVC. The symbol * indicates significant differences (*p* < .05) between MFGM and PLA groups

**FIGURE 4:**
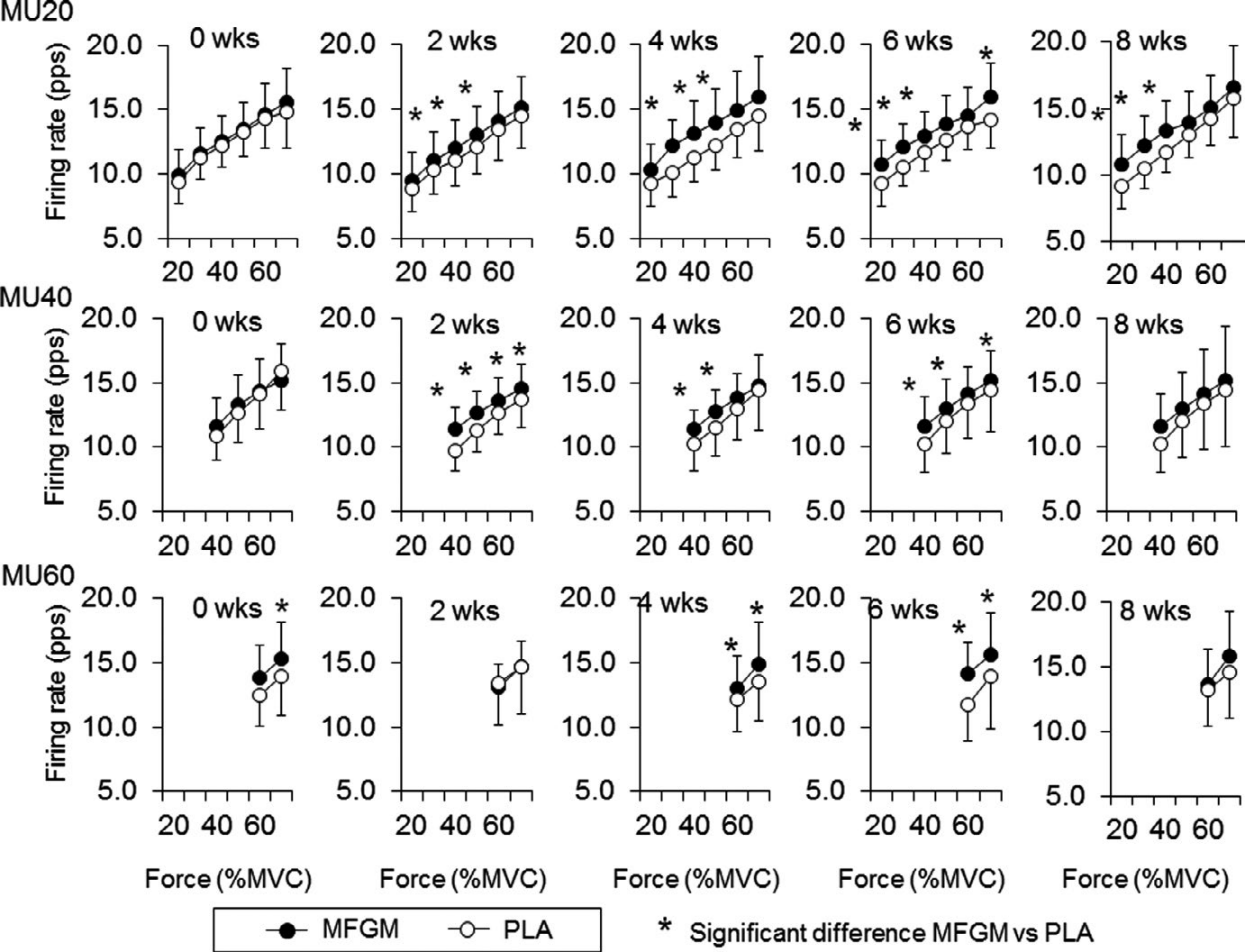
Comparisons of motor unit firing rates during Ramp70 between the groups with ingestions of milk fat globule membrane (MFGM; filled circles) and placebo (PLA; open circles). MU20: motor units recruited at <20% of maximal voluntary contraction (MVC), MU40: motor units recruited at 20%–40% of MVC, MU60: motor units recruited at 40%–60% of MVC. The symbol * indicates significant differences (*p* < .05) between MFGM and PLA groups

At 0 weeks, significant higher firing rates in MFGM when comparing with PLA were observed in MU10 at 25% of MVC (11.9 ± 1.7 pps for MFGM and 11.0 ± 1.8 pps for PLA, *p* = .027) and in MU30 at 30% of MVC (10.5 ± 1.8 pps for MFGM and 9.6 ± 1.6 pps for PLA, *p* = .007) for Ramp30 (Figure 3) and in MU60 at 70% of MVC (15.3 ± 2.9 pps for MFGM and 13.9 ± 3.0 pps for PLA, *p* = .026) for Ramp70 (Figure 4). At 2 weeks, significant higher firing rates in MFGM when comparing with PLA were observed in MU10 at 10% (9.7 ± 2.3 pps for MFGM and 8.6 ± 1.6 pps for PLA, *p* = .031), 15% (11.5 ± 1.8 pps for MFGM and 9.4 ± 1.6 pps for PLA, *p* = .001), 20% (12.3 ± 1.6 pps for MFGM and 10.1 ± 1.9 pps for PLA, *p* = .001), 25% (12.5 ± 1.6 pps for MFGM and 10.5 ± 1.8 pps for PLA, *p* = .001), and 30% of MVC (12.6 ± 1.8 pps for MFGM and 10.7 ± 2.0 pps for PLA, *p* = .001), in MU20 at 20% (10.3 ± 1.6 pps for MFGM and 9.1 ± 1.9 pps for PLA, *p* = .001), 25% (11.3 ± 1.5 pps for MFGM and 9.9 ± 1.9 pps for PLA, *p* = .001), and 30% of MVC (11.3 ± 1.4 pps for MFGM and 10.2 ± 2.0 pps for PLA, *p* = .001) and in MU30 at 30% of MVC (10.2 ± 1.4 pps for MFGM and 9.4 ± 1.6 pps for PLA, *p* = .010) for Ramp30 (Figure 3) and in MU20 at 30% (11.1 ± 2.1 pps for MFGM and 10.3 ± 1.9 pps for PLA, *p* = .022), 40% (12.0 ± 2.1 pps for MFGM and 11.1 ± 2.0 pps for PLA, *p* = .004), and 50% of MVC (13.0 ± 2.2 pps for MFGM and 12.1 ± 2.1 pps for PLA, *p* = .006) and in MU40 at 40% (11.4 ± 1.7 pps for MFGM and 9.8 ± 1.5 pps for PLA, *p* = .001), 50% (12.7 ± 1.6 pps for MFGM and 11.3 ± 1.7 pps for PLA, *p* = .001), 60% (13.6 ± 1.7 pps for MFGM and 12.6 ± 1.7 pps for PLA, *p* = .007), and 70% of MVC (14.5 ± 1.8 pps for MFGM and 13.7 ± 2.2 pps for PLA, *p* = .019) for Ramp70 (Figure 4). At 4, 6, and 8 weeks, same trends of significant differences in motor unit firing rates between the MFGM and PLA groups (Figures 3 and 4).

**FIGURE 5:**
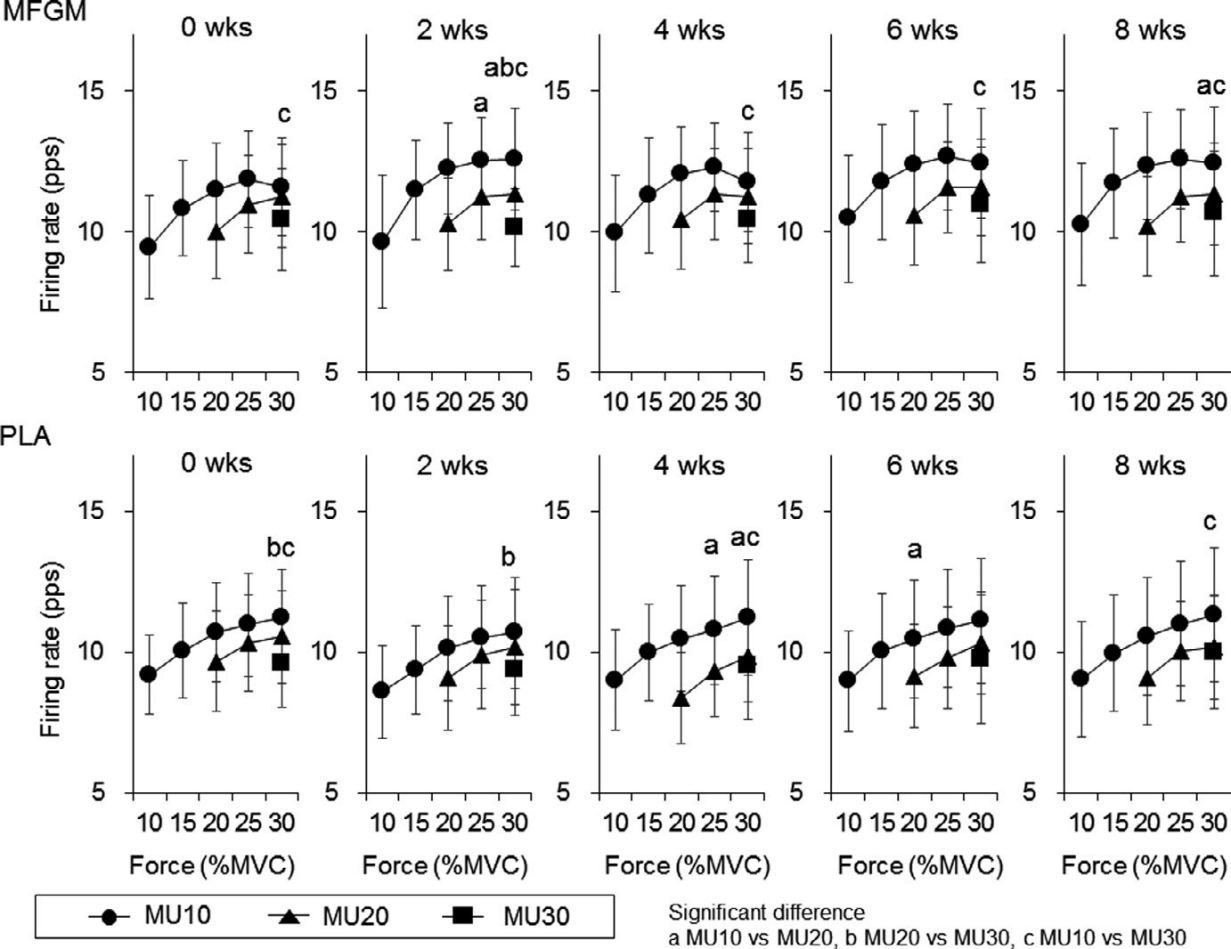
Comparisons of motor unit firing rates during Ramp30 among motor units with different recruitment thresholds for the groups with ingestions of milk fat globule membrane (MFGM; upper panels) and placebo (PLA; lower panels). MU10: motor units recruited at <10% of maximal voluntary contraction (MVC), MU20: motor units recruited at 10%–20% of MVC, MU30: motor units recruited at 20%–30% of MVC. The symbols a, b, and c indicate significant differences (*p* < .05) between MU10 and MU20, MU20 and MU30, and MU10 and MU30, respectively

**FIGURE 6:**
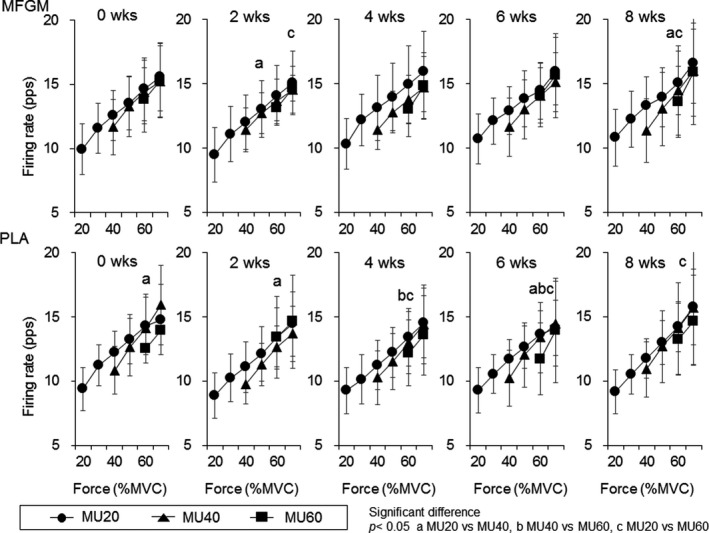
Comparisons of motor unit firing rates during Ramp70 among motor units with different recruitment thresholds for the groups with ingestions of milk fat globule membrane (MFGM; upper panels) and placebo (PLA; lower panels). MU20: motor units recruited at <20% of maximal voluntary contraction (MVC), MU40: motor units recruited at 20%–40% of MVC, MU60: motor units recruited at 40%–60% of MVC. The symbols a, b, and c indicate significant differences (*p* < .05) between MU20 and MU40, MU40 and MU60, and MU20 and MU60, respectively

For MFGM, firing rates of MU10 were significantly higher than those of MU30 at 30% of MVC at 0, 2, 4, 6, and 8 weeks (*p* = .004, .001, .010, .005, and .001) and were significantly higher than those of MU20 at 25% and 30% of MVC at 2 weeks (*p* = .024 and .007) and at 30% of MVC at 8 weeks (*p* = .010) and firing rate of MU20 were significantly higher than those of MU30 at 30% of MVC at 2 weeks (*p* = .015) for Ramp30 (Figure 5). For PLA, firing rate of MU10 were significantly higher than those of MU30 at 30% of MVC at 0, 4, and 8 weeks (*p* = .001, .001, and .013) and were significantly higher than those of MU20 at 25% and 30% of MVC at 4 weeks (*p* = .012 and .006) and firing rate of MU20 were significantly higher than those of MU30 at 30% of MVC at 0 and 2 weeks (*p* = .009 and .004) for Ramp30 (Figure 5). For MFGM, firing rates of MU20 were significantly higher than those of MU40 at 50% of MVC at 2 weeks (*p* = .007) and at 60% of MVC at 8 weeks (*p* = .002) and were significantly higher than those of MU60 at 70% of MVC at 2 weeks (*p* = .010) and at 60% of MVC at 8 weeks (*p* = .004) for Ramp70 (Figure 6). For PLA, firing rates of MU20 were significantly higher than those of MU40 at 60% of MVC at 0, 2, and 6 weeks (*p* = .001, .005, and .006) and were significantly higher than those of MU60 at 60% of MVC at 4, 6, and 8 weeks (*p* = .003, .001, and .015) and firing rates of MU40 were significantly higher than those of MU60 at 60% of MVC at 4 and 6 weeks (*p* = .008 and .004) for Ramp70 (Figure 6).

The average values and standard deviations (range) of power analysis for comparisons between groups at each period were 95.7 ± 1.7 (92.9–97.3) for MVC, 81.6 ± 9.4 (70.5–91.5) for RFD, 88.8 ± 5.3 (80.0–93.7) for muscle thickness, 89.0 ± 0.7 (88.2–89.7) for body mass, 94.8 ± 0.2 (94.5–94.9) for whole body muscle mass, 93.6 ± 0.7 (94.2–92.9) for lower limb muscle mass, 90.9 ± 8.2 (73.6–97.0) for nutritional parameters, 87.1 ± 14.4 (60.6–100.0) for firing rate of motor units recruited at <10% of MVC during Ramp30, 88.0 ± 15.4 (52.3–100.0) for firing rate of motor units recruited at 10%–20% of MVC during Ramp30, 71.8 ± 4.5 (66.7–78.1) for firing rate of motor units recruited at 20%–30% of MVC during Ramp30, 79.6 ± 16.3 (50.3–99.9) for firing rate of motor units recruited at <20% of MVC during Ramp70, 80.1 ± 15.8 (50.3–99.8) for firing rate of motor units recruited at 20%–40% of MVC during Ramp70, and 74.6 ± 17.9 (57.6–99.2) for firing rate of motor units recruited at 40%–60% of MVC during Ramp70. The average values and standard deviations (range) of power analysis for comparisons among periods for each group were 90.4 ± 5.3 (84.2–95.9) for MVC, 93.0 ± 5.0 (82.0–97.3) for RFD, 95.5 ± 2.1 (92.1–97.5) for muscle thickness, 96.2 ± 1.6 (94.1–97.5) for body mass, 97.0 ± 0.2 (96.8–97.2) for whole body muscle mass, 97.2 ± 0.3 (96.8–97.5) for lower limb muscle mass, 87.6 ± 11.0 (59.3–97.2) for nutritional parameters, 79.6 ± 15.8 (50.9–97.4) for firing rate of motor units recruited at <10% of MVC during Ramp30, 81.7 ± 12.0 (53.7–97.4) for firing rate of motor units recruited at 10%–20% of MVC during Ramp30, 88.0 ± 10.1 (67.1–97.2) for firing rate of motor units recruited at 20%–30% of MVC during Ramp30, 77.8 ± 12.6 (53.5–96.2) for firing rate of motor units recruited at <20% of MVC during Ramp70, 81.8 ± 13.8 (51.4–97.4) for firing rate of motor units recruited at 20%–40% of MVC during Ramp70, and 83.1 ± 10.3 (61.8–97.5) for firing rate of motor units recruited at 40%–60% of MVC during Ramp70.

## 4 DISCUSSION

This study examined the effect of MFGM supplementation on the motor unit firing pattern following 8 weeks of resistance training in older adults. We found no significant differences in muscle thickness or maximal muscle strength between the two groups, significant differences in motor unit firing rates at 2, 4, 6, and 8 weeks after the intervention between the two groups, and different time‐courses in changes of relationships in firing rates among different motor unit groups with different recruitment thresholds between the two groups. These results do not support a part of our hypotheses that MFGM ingestion induces an increase in motor unit firing rates at the same absolute force and then changes adaptations in the motor unit firing pattern during the intervention of resistance training.

We applied three sets of five isometric contractions at greater than 80% of MVC twice a week for 8 weeks to both MFGM and PLA groups in this study. This training regimen may be of a higher intensity and lower volume when compared with those widely used and recommended for older adults (Fragala et al., 2019). Our previous study used double the training volume applied in the present study, that is, 3 sets of 10 isometric contractions at greater than 80% of MVC twice a week for 8 weeks, inducing an increase of 24.7%/ 8 weeks in older adults (Watanabe et al., 2019). In the present study, PLA showed a significant increase in MVC at 4, 6, and 8 weeks when compared with 0 weeks (*p* < .05), while no significant changes were observed in MVC for the MFGM group during the intervention (*p* > .05). Rates of MVC increase in MFGM and PLA groups from 0 to 8 weeks were 6.1 and 18.7%/8 weeks, respectively. Although rates of increase were lower, a detectable training effect on muscle strength was confirmed in PLA, which employed higher‐intensity and lower‐volume resistance training. However, inter‐individual differences in muscle strength gain are greater in the training regimen employed in this study. This could be the reason why we did not detect a significant increase in MVC for the MFGM group during the intervention. Also, we detected the trend of a lower RFD (Table 2) and greater physical activity (Table 4) in the PLA group. Although we could not detect any relationships between these parameters and MVC such as in correlation analysis, trainability of the force production capacity and/or physical activity in daily life may partly explain the significant increase in MVC only in the PLA group. Whole body and lower extremity muscle mass and muscle thickness, which are indicators of muscular adaptations, were not significantly changed during the intervention. These results are similar to those of our previous study using double the training volume (Watanabe et al., 2019).

When comparing motor unit firing rates between the groups, the MFGM group showed greater firing rates than the PLA group after 2 weeks of intervention for Rmap30 and Ramp70 (*p* < .05; Figures 3 and 4). These differences may be explained by a different trend of changes in motor unit firing rates during the intervention between the MFGM and PLA groups. Motor unit firing rates in the MFGM group tended to gradually increase following the intervention, while significant differences were not detected (Figures 1 and 2). Recent studies using high‐density surface EMG showed that resistance training induces an increase in the motor unit firing rate during submaximal contractions (Del Vecchio et al., 2019; Vila‐Cha et al., 2010), and suggest that this adaptation is elicited by an increase in the net excitability input to the motor neuron pool for the same relative forces. Therefore, we consider that adaptations in the MFGM group are in line with adaptations that are generally recognized as motor unit adaptations following resistance training. The absence of significant differences in the increase in the motor unit firing rate after the intervention in the MFGM group could be explained by the relatively lower training volume in this study. On the other hand, the motor unit firing rate in the PLA group tended to decrease from 0 to 4 or 6 weeks during the intervention, and parts showed significant differences (Figures 1 and 2). While an increase or no change in the motor unit firing rate following resistance training was reported in most previous studies, Pucci et al. reported a decrease in the motor unit firing rate at the same absolute force level during 3 weeks of isometric training of the quadriceps femoris muscles in young adults (Pucci, Griffin, & Cafarelli, 2006). They suggested that a leftward shift in the force‐firing rate curve was a cause of a decrease in the motor unit firing rate at absolute forces. In fact, the PLA group showed a significant increase in MVC after 4 weeks of intervention (*p* < .05; Table 2), and the force‐firing rate curve in that group should show a leftward shift. From these results, opposite changes between the MFGM and PLA groups in motor unit firing rates during the intervention would be the main cause of significant differences in motor unit firing rates between them after 2 weeks of intervention (Figures 3 and 4). We also detected differences in changes in the motor unit firing pattern between MFGM and PLA groups based on relationships of the firing rate among motor unit groups with different recruitment thresholds (Figures 5 and 6). In order to quantify changes in the motor unit firing pattern, we counted the pairs of motor unit groups showing significant differences in the firing rate at each force level, and interpreted changes in the number of pairs as changes in motor unit firing patterns (Watanabe et al., 2019). We already reported that significant differences in firing rates among motor unit groups with different recruitment thresholds are present in young but not older adults (Watanabe et al., 2016), and that these significant differences can be induced by resistance training intervention in older adults (Watanabe et al., 2018, 2019). The numbers of pairs of motor unit groups showing a significant difference increased from 0 to 2 weeks in the MFGM group and from 2 to 4 weeks in the PLA group for both Ramp 30 and Ramp70 (Figures 5 and 6). These results can be interpreted as follows: a change in the motor unit firing pattern in the MFGM group occurs in an earlier phase of intervention than in the PLA group. Also, since an increase in the motor unit firing rate during resistance training intervention has been reported as a general neural adaptation, MFGM ingestion may induce neural adaptation even with a relatively lower training volume that cannot increase the motor unit firing rate with PLA.

Variations in changes in the motor unit firing pattern following resistance training have been identified in previous studies, and these would be due to differences in the training regimen, such as intensity, frequency, volume, and contraction mode (Duchateau, Semmler, & Enoka, 2006; Gabriel, Kamen, & Frost, 2006). Since this study used the same training regimen for the two groups, differences in changes in the motor unit firing rate may be mainly caused by nutritional supplementation. Our previous studies already identified alterations in motor unit adaptation following resistance training induced by nutritional supplementation (Watanabe et al., 2018, 2019). During 8 weeks of resistance training intervention, while significant increases in the muscle volume were found in older adults with fish protein ingestion, no significant changes in the muscle volume and marked changes in motor unit firing patterns were noted in older adults without fish protein ingestion (Watanabe et al., 2019). This previous study suggested that nutritional supplementation could separately modulate neural and muscular adaptations following resistance training. Using MFGM ingestion, Ota et al. (2015), Soga et al. (2015), and Minegishi et al. (2016) showed that nutritional supplementation induces a greater increase in motor functions following exercise intervention for older adults (Minegishi et al., 2016; Ota et al., 2015; Soga et al., 2015). They also noted marked changes in electrophysiological parameters such as the conduction velocity and amplitude in surface EMG on MFGM ingestion. These results suggest that improvement of motor functions by MFGM ingestion can be partly explained by neural adaptations, and this possibility may be supported by previous studies using mice. Oshida et al. (2003) and Yano et al. (2017) reported that MFGM and its major component (sphingomyelin) contribute to repairing denervation or demyelination at neuromuscular junctions in aged mice (Oshida et al., 2003; Yano et al., 2017). Although the present study did not involve the measurement of any physiological responses associated with neuromuscular junctions, different changes in the motor unit firing pattern between MFGM and PLA groups could be partly explained by alterations in the neuromuscular system induced by MFGM ingestion that are noted in animal studies. Also, we did not detect increases in MVC in MFGM and significant differences in MVC and RFD between MFGM and PLA, although MFGM and PLA showed different motor unit adaptations. Due to methodological limitations, we only used submaximal contraction to record multi‐channel surface EMG. We thus assumed that differences in the performed force levels could explain the inconsistent results between motor function test such as MVC or RFD that require maximal contraction and motor unit firing patterns that required submaximal contraction.

It should be noted that this study was performed without sample size calculation before the experiment and statistical power was confirmed by a postanalysis statistical power analysis. Since many studies set a power at 80% (0.80; Hulley, Cummings, Browner, Grady, & Newman, 2013) and the mean values of statistical powers of this study were greater than 80%, statistical powers of this study could be sufficient to analyze the observed results. It is thought that the results of power analysis in percentage mean chance of rejecting the null hypothesis (Vincent, 2005). We thus interpreted that statistical powers in our study were enough to compare the variables between groups and among the periods.

We applied combination of resistance training and nutritional supplementations to older adults for testing the effect of MFGM ingestion on resistance training‐induced motor unit adaptations. To clarify the effect of MFGM ingestion, physical activity level, and nutritional condition in daily life during the intervention should be controlled to minimize the effects other than the resistance training and MFGM ingestion. Physiological adaptation may be induced by parts of physical activity and daily meals. We detected greater physical activity levels in PLA while statistical significances were not observed and large inter‐individual variations in physical activity level in each group (Table 4). Since we provided high‐intensity training that the participants would not experience in normal daily life even if they have exercise habits, we would be able to conclude that the training regimen in the present study is specific stimulus for the participants’ neuromuscular systems and induces training‐specific physiological adaptations in all participants. Moreover, we should note that MFGM showed significant increases in total energy and carbohydrate consumption during the intervention (Table 3). Daily meals would be also important factors for exercise‐induced physiological adaptations. To our knowledge, no studies reported that total energy and carbohydrate consumption change neural adaptations such as motor unit firing patterns during resistance training. These variations in physical activity level and nutritional condition between the groups should be noted when the results of our study are interpreted. Further work with more controlled conditions would be necessary to clarify the detailed functions of MFGM ingestion during training intervention.

## 5 CONCLUSION

We investigated the effect of MFGM supplementation on motor unit adaptation following resistance training in 24 older adults. During 8 weeks of intervention: there were no significant differences in muscle thickness or maximal muscle strength between the two groups (*p* > .05); there were significant differences in motor unit firing rates at 2, 4, 6, and 8 weeks after the intervention between the two groups (*p* < .05); and the time‐course of changes in relationships of the firing rate among different motor unit groups with different recruitment thresholds differed between the two groups (*p* < .05). These results suggest that MFGM supplementation induces different motor unit adaptation in older adults.

## CONFLICT OF INTEREST

The authors have no conflicts of interest related to the study.

## AUTHOR CONTRIBUTION

KW: Planning research, conducting experiment, analyzing data, discussing results, and writing paper. AH: Analyzing data, discussing results, and editing and reviewing manuscript. AT: Planning research, conducting experiment, analyzing data, discussing results, and editing and reviewing manuscript. YM: planning research, analyzing data, discussing results, and editing and reviewing manuscript.
